# Effect of once-daily ICS/LAMA/LABA triple therapy versus ICS/LABA on respiratory symptoms (E-RS: Asthma): Analysis of the phase IIIA CAPTAIN trial

**DOI:** 10.1016/j.jacig.2026.100741

**Published:** 2026-06-01

**Authors:** Emilio Pizzichini, Guy Brusselle, Jodie Crawford, Hiromasa Inoue, Huib A.M. Kerstjens, John Oppenheimer, Alberto Papi, Ian D. Pavord, David Slade, Liza Yuanita, Alison Moore

**Affiliations:** a GSK, Brentford, Middlesex, United Kingdom; b Universidade Federal de Santa Catarina, Florianopolis, Brazil; c Department of Respiratory Medicine, Ghent University Hospital, Ghent, Belgium; d Development Biostatistics, GSK, London, United Kingdom; e Department of Pulmonary Medicine, Graduate School of Medical and Dental Sciences, Kagoshima University, Kagoshima, Japan; f Groningen Research Institute for Asthma and COPD, University of Groningen and University Medical Center Groningen, Groningen, The Netherlands; g Rutgers New Jersey Medical School, Newark, NJ; h University of Ferrara, Ferrara, Italy; i Oxford Respiratory NIHR BRC, University of Oxford, Oxford, United Kingdom; j GSK, Research Triangle Park, NC; k Medical Affairs Asthma & COPD, GSK, Minato-ku, Tokyo, Japan; l Global Medical Affairs, General Medicines, GSK, London, United Kingdom

**Keywords:** Asthma, triple therapy, symptoms, Evaluating Respiratory Symptoms, long-acting muscarinic antagonist, inhaled corticosteroid, long-acting β_2_-agonist, umeclidinium, fluticasone furoate, vilanterol

## Abstract

**Background:**

Addition of the long-acting muscarinic antagonist umeclidinium (UMEC) to the inhaled corticosteroid/long-acting β_2_-agonist (ICS/LABA) combination fluticasone furoate/vilanterol (FF/VI) improved lung function in adults with uncontrolled moderate to severe asthma in the CAPTAIN (Clinical study of Asthma Patients receiving Triple therapy through A single INhaler) study; however, the impact on symptoms requires further investigation.

**Objective:**

We sought to evaluate the effect of adding UMEC to FF/VI on asthma symptoms.

**Methods:**

The CAPTAIN study was a phase IIIA, randomized, controlled, 24- to 52-week study of patients with uncontrolled moderate to severe asthma despite ICS/LABA receiving once-daily single-inhaler FF/VI (100/25 or 200/25 μg) or FF/UMEC/VI (100/31.25/25, 100/62.5/25, 200/31.25/25, or 200/62.5/25 μg). Here, we compare the effect of pooled FF/UMEC 62.5/VI (100/62.5/25 and 200/62.5/25 μg) versus FF/VI (100/25 and 200/25 μg) on symptom control using prespecified analyses of change from baseline in Evaluating Respiratory Symptoms in Asthma (E-RS: Asthma) total and domain scores, and proportion of patients meeting an E-RS: Asthma total score responder threshold. We also performed *post hoc* analyses assessing the impact of baseline type 2 inflammation status on treatment response.

**Results:**

Least-squares mean (95% CI) reductions from baseline in E-RS: Asthma total score exceeded the minimum clinically important difference (−2.0 units) and were numerically greater with FF/UMEC 62.5/VI (−2.89 [−3.15 to −2.64]; n = 814) versus FF/VI (−2.47 [−2.73 to −2.22]; n = 813). The proportion of responders (45% [n = 360] vs 41% [n = 327]) and odds of response (odds ratio, 1.18 [95% CI, 0.96 to 1.45]) were numerically greater with FF/UMEC 62.5/VI versus FF/VI. Similar trends were observed irrespective of type 2 status.

**Conclusions:**

Patients with symptomatic asthma may benefit from optimized treatment interventions, such as adding a long-acting muscarinic antagonist to ICS/LABA.

Asthma is characterized by symptoms including shortness of breath, cough, and chest tightness as well as signs of variable airflow limitation.^1^ The control of asthma symptoms has an impact on the burden of disease, with worse control associated with poorer health-related quality of life, more frequent health care resource utilization, and worse disease outcomes.^2^, ^3^, ^4^ The 2025 Global INitiative for Asthma (GINA) report recommends assessing symptom control at every opportunity and highlights the achievement of good symptom control as a key treatment goal.^1^ For patients with uncontrolled asthma despite medium- or high-dose inhaled corticosteroid/long-acting β_2_-agonist (ICS/LABA) therapy (ie, GINA steps 4 and 5), the addition of a long-acting muscarinic antagonist (LAMA) as triple therapy is recommended.^1^ Other national and international asthma management guidelines also recommend the addition of a LAMA to ICS/LABA therapy in these patient groups.^5^, ^6^, ^7^, ^8^

The CAPTAIN (Clinical study of Asthma Patients receiving Triple therapy through A single INhaler) study was a randomized phase IIIA trial that investigated the effect of adding the LAMA umeclidinium (UMEC) to the ICS/LABA combination of fluticasone furoate/vilanterol (FF/VI) in a single inhaler, or doubling FF dose, in adults with uncontrolled moderate to severe asthma despite ICS/LABA therapy.^9^ Patients included in the CAPTAIN trial were symptomatic but were not required to have experienced an exacerbation in the previous year, thus improving the generalizability of results to real-world patients,^9^ who typically experience a low rate of exacerbations.^10^^,^^11^ The addition of UMEC to FF/VI led to significant improvements in lung function as well as numerical improvements in symptom control (7-item Asthma Control Questionnaire [ACQ-7] responder rates) and moderate/severe exacerbation rates.^9^ Furthermore, increasing the FF dose led to numerical reductions in the rate of moderate/severe exacerbations, particularly in patients with high type 2 (T2) inflammation status.^9^ These findings suggest that patients with uncontrolled moderate to severe asthma may benefit from treatment optimization based on T2 phenotype and that precision medicine is relevant for this patient group.

Symptom control is a key component of asthma assessment, alongside lung function and risk of exacerbations.^1^ However, there is limited evidence showing a correlation between symptom tools and other objective measures of asthma, such as spirometry.^12^, ^13^, ^14^ Further investigation into the impact of therapy on symptoms is therefore of clinical interest. In the CAPTAIN trial, symptom control was assessed via multiple prespecified patient-reported outcomes, including the Evaluating Respiratory Symptoms in Asthma (E-RS: Asthma) tool.^9^ The E-RS: Asthma is an 11-item patient-reported daily diary derived from the Evaluating Respiratory Symptoms in Chronic Obstructive Respiratory Disease (E-RS: COPD) tool^15^, ^16^, ^17^ and has been shown to be reliable and responsive in assessing respiratory symptoms in patients with moderate to severe asthma.^15^ The E-RS: Asthma captures a broader range of respiratory symptoms than the ACQ, including cough, and usefully categorizes symptoms into specific domains; however, reports of its use in the published literature are limited.^15^^,^^18^ Consequently, herein we report the effect of adding UMEC to FF/VI on patients’ asthma symptom burden using the prespecified E-RS: Asthma secondary end point from the CAPTAIN study. Additional *post hoc* analyses assessed the impact of treatment on symptom control in patients according to T2 status.

## Methods

### Study design

The CAPTAIN trial was a phase IIIA, double-blind, randomized, controlled, 24- to 52-week, parallel-group study (GSK study no. 205715; NCT02924688). Full details of the CAPTAIN study have been reported elsewhere.^9^ Briefly, patients received twice-daily, open-label fluticasone propionate plus salmeterol (FP/SAL) 250/50 μg via the DISKUS dry-powder inhaler (DPI; GSK) for a 3-week run-in period, followed by open-label FF/VI 100/25 μg via the ELLIPTA DPI (GSK) during the subsequent 2-week stabilization period. Patients were randomly assigned (1:1:1:1:1:1) to 1 of 6 treatment groups: once-daily FF/VI 100/25 or 200/25 μg, or FF/UMEC/VI 100/31.25/25, 100/62.5/25, 200/31.25/25, or 200/62.5/25 μg (ELLIPTA DPI).

The CAPTAIN study was performed in accordance with the Declaration of Helsinki, International Conference on Harmonisation Good Clinical Practice, and applicable country-specific regulatory requirements and was approved by applicable central or local institutional review boards or independent ethics committees.

### Study population

Patients eligible for inclusion in the CAPTAIN study were 18 years or older with diagnosed asthma and poor symptom control (ACQ-6 score ≥1.5) despite receiving daily ICS/LABA maintenance therapy (daily FP >250 μg or equivalent) for 12 weeks or more before prescreening, with no changes to therapy in the 6 weeks before prescreening. Spirometry was carried out at all clinic visits including screening, enrollment, and randomization; reversibility was assessed at screening. Patients had a prebronchodilator FEV_1_ of 30% or higher and less than 85% of the predicted normal value and displayed reversibility to salbutamol/albuterol (a postbronchodilator ≥12% and ≥200 mL increase in FEV_1_), both at screening. A documented asthma-related health care contact or temporary change in asthma therapy for treatment of acute asthma symptoms was also necessary in the year before screening. Patients were not required to have had a severe exacerbation in the previous year and were excluded from the study if they had experienced an asthma exacerbation requiring a change in asthma maintenance therapy in the 6 weeks before screening. Patients were also excluded if they had COPD or concurrent respiratory disorders including pneumonia or they were current smokers or had a history of smoking (≥10 pack years).

### Analysis end points and assessments

The primary end point of the CAPTAIN study was change from baseline in clinic trough FEV_1_ at week 24, for which results have been previously reported.^9^ The present analysis evaluated data from a prespecified secondary end point of the CAPTAIN trial, the E-RS: Asthma, to assess the effect on patients’ symptoms following treatment with FF/UMEC 62.5/VI (UMEC 62.5 μg) or FF/VI. The E-RS: Asthma tool captures self-reported respiratory symptoms over the previous 24 hours, assessed via 11 items across 3 symptom domains: RS-Breathlessness (5 items: “breathless today,” “breathless with activity,” “short of breath—personal care,” “short of breath—indoor activity,” and “short of breath—outdoor activity”; score range, 0-17), RS-Chest Symptoms (3 items: “congestion,” “discomfort,” and “tightness”; score range, 0-12), and RS-Cough and Sputum (3 items: “cough frequency,” “mucus quantity,” and “difficulty with mucus”; score range, 0-11).^15^ An E-RS: Asthma total score is summed ranging from 0 to 40; higher scores represent greater symptom burden.^15^

Data were analyzed for the total and domain scores as least-squares (LS) mean (95% CI) change from baseline at weeks 21 to 24, and for the proportion of responders, defined as those achieving the minimum clinically important difference (MCID) for improvement in E-RS: Asthma total score change greater than or equal to 2.0 units (decrease) from baseline at weeks 21 to 24. Nonresponders were defined as those having an increase, no change, or a decrease from baseline of less than 2 points or a missing E-RS: Asthma total score at the postbaseline visit. Patients completed the E-RS: Asthma questionnaire at home using an eDiary during the run-in period of the CAPTAIN study and throughout the study; data were analyzed at 4-weekly intervals. Meaningful change thresholds for the E-RS: Asthma have been previously defined as follows: RS-Total, −2.0; RS-Breathlessness, −1.0; RS-Cough and Sputum, −0.7; and RS-Chest Symptoms, −0.7.^15^

### Statistical analysis

**Fig 1:**
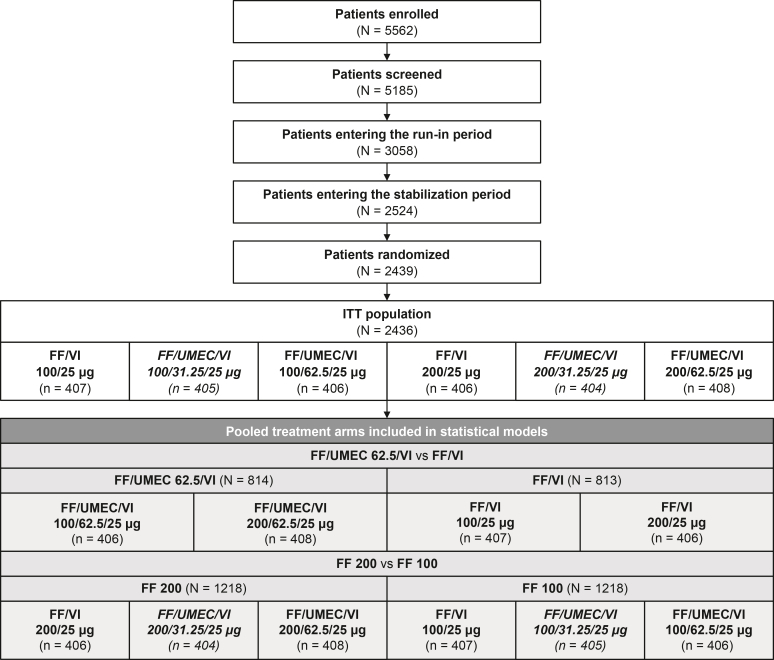
Patient flow.

The primary CAPTAIN analysis assessed the clinical benefit of adding UMEC to FF/VI. As per the prespecified analysis plan, to enhance the power and precision of analyses, in the statistical analysis, FF/UMEC/VI data were pooled such that UMEC doses could be evaluated separately (FF/UMEC 31.25/VI [100/31.25/25 and 200/31.25/25 μg] and FF/UMEC 62.5/VI [100/62.5/25 and 200/62.5/25 μg]) and compared with pooled FF/VI (100/25 and 200/25 μg). However, here, wherein we report results from prespecified pooled analyses of the E-RS: Asthma secondary end point, we present only the FF/UMEC 62.5/VI (100/62.5/25 and 200/62.5/25 μg) versus FF/VI (100/25 and 200/25 μg) treatment comparison, because this is the UMEC dose selected for approved FF/UMEC/VI combinations (Fig 1).^19^^,^^20^ We also performed analyses of the E-RS: Asthma end point using data unpooled by the FF dose: FF/UMEC/VI 100/62.5/25 and 200/62.5/25 μg and FF/VI 100/25 and 200/25 μg. Unpooled analyses of E-RS: Asthma total scores were prespecified, whereas analyses of individual domains were *post hoc*.

Because a step-down closed testing approach was used to control for multiplicity in the CAPTAIN study, and statistical significance was not shown for the key secondary end point (annualized rate of moderate/severe exacerbations), statistical inferences could not be made for subsequent tests in the hierarchy, including the E-RS: Asthma. Therefore, data were reported descriptively.

Change from baseline in E-RS: Asthma total and domain scores for pooled and unpooled treatment groups were analyzed using a mixed-model repeated-measures model, including covariates of treatment group, sex, region, 4-weekly period, prestudy ICS dosage at screening, age, baseline value, and interactions of baseline value by 4-weekly period and treatment group by 4-weekly period. The analysis and estimand of interest was based on the intention-to-treat (ITT) population and used a treatment policy estimand strategy, estimating the treatment effect regardless of randomized treatment discontinuation (ie, using on-treatment and posttreatment data up to and including week 24). The proportion of responders was analyzed using logistic regression models on the ITT population and also used a treatment policy estimand strategy, estimating the treatment effect regardless of randomized treatment discontinuation.

Additional *post hoc* analyses explored the impact of adding UMEC 62.5 μg to FF/VI or increasing the FF dose (FF 200 [pooled FF/VI 200/25; FF/UMEC/VI 200/31.25/25 and 200/62.5/25] vs FF 100 [pooled FF/VI 100/25; FF/UMEC/VI 100/31.25/25 and 100/62.5/25]) (Fig 1) on the E-RS: Asthma end point according to baseline eosinophil (EOS) levels, fractional exhaled nitric oxide (Feno) levels, and T2 status (EOS and Feno combined). As per the original CAPTAIN analysis,^9^ baseline T2 biomarkers were categorized as EOS low (<150 cells/μL), intermediate (150 to <300 cells/μL), and high (≥300 cells/μL); Feno low (<20 parts per billion [ppb]), intermediate (20 to ≤50 ppb), and high (>50 ppb); and combined T2 low (EOS <150 cells/μL and Feno <20 ppb), intermediate (all other patients with an EOS and Feno measurement), and high (EOS ≥300 cells/μL and Feno >50 ppb). Pooled and unpooled data were analyzed using a mixed-model repeated-measures model with covariates of treatment group, sex, region, 4-weekly period, age, baseline value, prestudy ICS dosage at screening, EOS and/or Feno category, interactions of baseline value by 4-weekly period, treatment group by 4-weekly period, EOS and/or Feno category by treatment group, EOS and/or Feno category by 4-weekly period, and EOS and/or Feno category by treatment group and 4-weekly period.

## Results

### Baseline demographic and clinical characteristics

In total, 2436 patients were included in the CAPTAIN ITT population. For the prespecified pooled analyses assessing the effect of adding UMEC to FF/VI, 814 patients treated with FF/UMEC/VI 100/62.5/25 (n = 406) or 200/62.5/25 (n = 408) were included in the pooled FF/UMEC 62.5/VI group and 813 patients treated with FF/VI 100/25 (n = 407) or 200/25 (n = 406) were included in the pooled FF/VI group (Fig 1).

For *post hoc* analyses assessing the effect of increasing ICS dose by T2 status, the pooled FF 100 group (n = 1218) included FF/UMEC/VI 100/62.5/25 and FF/VI 100/25, as well as 405 patients treated with FF/UMEC/VI 100/31.25/25; the pooled FF 200 group (n = 1218) included FF/UMEC/VI 200/62.5/25 and FF/VI 200/25, as well as 404 patients treated with FF/UMEC/VI 100/31.25/25 (Fig 1). The treatment comparison for FF/UMEC 31.25/VI versus FF/VI is not presented here for reasons stated earlier.

Baseline demographic and clinical characteristics for the pooled FF/VI and FF/UMEC 62.5/VI groups were generally similar; mean ± SD E-RS: Asthma total scores at baseline were low for both FF/VI (8.16 ± 6.17) and FF/UMEC 62.5/VI (8.35 ± 6.07) (Table I). Baseline characteristics for the CAPTAIN ITT population have been reported previously.^9^

**Table I:** Baseline demographic and clinical characteristics for pooled FF/VI and FF/UMEC 62.5/VI treatment groups∗

Characteristics	FF/VI (N = 813)	FF/UMEC 62.5/VI (N = 814)	CAPTAIN ITT population (N = 2436)
*Demographic characteristics*			
Age (y), mean ± SD	53.6 ± 13.16	53.3 ± 12.95	53.2 ± 13.11
Male, n (%)	307 (38)	308 (38)	922 (38)
BMI (kg/m^2^), mean ± SD	29.3 ± 6.19	29.5 ± 6.79	
*Clinical characteristics*			
E-RS: Asthma score, mean ± SD†	**n = 805**	**n = 807**	**n = 2418**
Total score	8.16 ± 6.17	8.35 ± 6.07	8.30 ± 6.14
RS-Breathlessness score	3.90 ± 3.23	4.00 ± 3.17	3.94 ± 3.20
RS-Chest symptoms score	2.07 ± 1.84	2.12 ± 1.80	2.12 ± 1.81
RS-Cough and sputum score	2.19 ± 1.70	2.23 ± 1.69	2.23 ± 1.70
Total no. of exacerbations, n (%)‡			
0	124 (15)	107 (13)	364 (15)
1	470 (58)	469 (58)	1390 (57)
≥2	219 (27)	238 (29)	682 (28)
Total no. of exacerbations requiring OCS, SCS, and/or hospitalization, n (%)‡			
0	301 (37)	284 (35)	892 (37)
1	394 (48)	395 (49)	1166 (48)
≥2	118 (15)	135 (17)	378 (16)
Prebronchodilator FEV_1_ (mL), mean ± SD§	**n = 803** 1728 ± 591	**n = 811** 1744 ± 605	**n = 2423** 1734 ± 584
Prebronchodilator FEV_1_% predicted, mean ± SD§	**n = 803** 58.45 ± 13.12	**n = 811** 58.87 ± 12.99	**n = 2423** 58.48 ± 12.79
Postbronchodilator FEV_1_/FVC, mean ± SD§	**n = 811** 0.66 ± 0.11	**n = 811** 0.66 ± 0.11	**n = 2430** 0.66 ± 0.11
ACQ-7 score, mean ± SD‖	**n = 793** 2.13 ± 0.70	**n = 795** 2.10 ± 0.69	**n = 2383** 2.12 ± 0.70
Blood EOS§¶	**n = 792**	**n = 802**	**n = 2386**
Geometric mean (SD of log) (cells/μL)	227 (923.8)	230 (912.2)	228 (950.6)
EOS low (<150 cells/μL), n (%)	215 (27)	222 (28)	651 (27)
EOS intermediate (150 to <300 cells/μL), n (%)	252 (32)	220 (27)	697 (29)
EOS high (≥300 cells/μL), n (%)	325 (41)	360 (45)	1038 (44)
Feno‖¶	**n = 747**	**n = 757**	**n = 2262**
Geometric mean (SD of log) (ppb)	20.5 (0.65)	19.9 (0.67)	19.7 (0.66)
Feno low (<20 ppb), n (%)	376 (50)	400 (53)	1198 (53)
Feno intermediate (20 to ≤50 ppb), n (%)	296 (40)	287 (38)	855 (38)
Feno high (>50 ppb), n (%)	75 (10)	70 (9)	209 (9)
Combined T2 status (blood EOS§¶ and Feno‖¶)	**n = 727**	**n = 745**	**n = 2217**
T2 low (EOS <150 cells/μL and Feno <20 ppb), n (%)	119 (16)	133 (18)	405 (18)
T2 intermediate (all other patients with an EOS and Feno measurement), n (%)	565 (78)	568 (76)	1674 (76)
T2 high (EOS ≥300 cells/μL and Feno >50 ppb), n (%)	43 (6)	44 (6)	138 (6)

*BMI*, Body mass index; *FVC*, forced vital capacity; *OCS*, oral corticosteroid; *SCS*, systemic corticosteroid.

∗ Data of patients in the ITT population receiving UMEC 31.25 μg (n = 809) are not presented.

† Over the last 14 d before starting the randomized treatment.

‡ In the 12 mo before the screening visit.

§ At screening.

‖ At randomization.

¶ *Post hoc* analysis.

### Change from baseline in E-RS: Asthma scores at weeks 21 to 24 (prespecified analysis)

**Fig 2:**
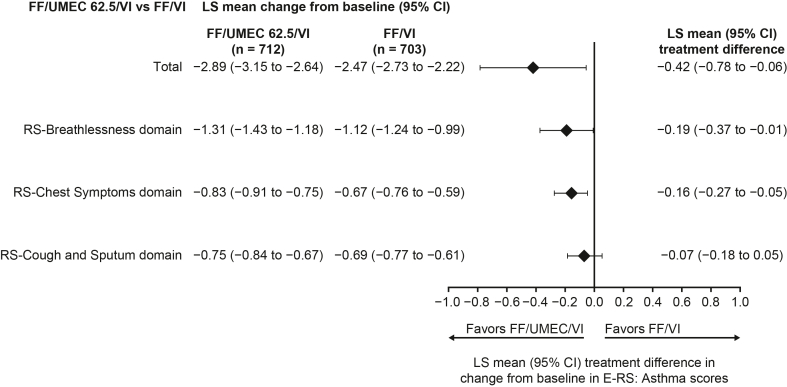
Pooled E-RS: Asthma total and domain scores at weeks 21 to 24.∗ Because of the break in hierarchy in the step-down closed testing approach used in the CAPTAIN study, wherein statistical significance was not shown for the key secondary end point, data for the E-RS: Asthma secondary end point are descriptive and statistical inference cannot be made. ∗Patients with analyzable data at weeks 21 to 24.

At weeks 21 to 24, asthma symptoms improved in both pooled FF/UMEC 62.5/VI and FF/VI treatment groups, as measured by a reduction in E-RS: Asthma total scores from baseline (Fig 2). The LS mean (95% CI) reduction in E-RS: Asthma total score from baseline exceeded the MCID (−2.0 units) and was numerically greater with FF/UMEC 62.5/VI (−2.89 [−3.15 to −2.64]) versus FF/VI (−2.47 [−2.73 to −2.22]) (Fig 2). The reductions in LS mean (95% CI) RS-Breathlessness (−1.31 [−1.43 to −1.18] vs −1.12 [−1.24 to −0.99]) and RS-Chest Symptoms (−0.83 [−0.91 to −0.75] vs −0.67 [−0.76 to −0.59]) domain scores were numerically greater with FF/UMEC 62.5/VI versus FF/VI; the RS-Cough and Sputum domain score was also improved with FF/UMEC 62.5/VI versus FF/VI (−0.75 [−0.84 to −0.67] vs −0.69 [−0.77 to −0.61]), although the difference was small (Fig 2).

Similar results were seen with unpooled data (analyses of E-RS: Asthma total scores were prespecified; analyses of individual domains were *post hoc*), with reductions in E-RS: Asthma total score from baseline exceeding the MCID (−2.0 units) in all treatment groups, and greater reductions were seen with FF/UMEC 62.5/VI versus FF/VI regardless of the FF dose (see Fig E1 in this article’s Online Repository at www.jaci-global.org).

### E-RS: Asthma responders (prespecified analysis)

**Fig 3:**
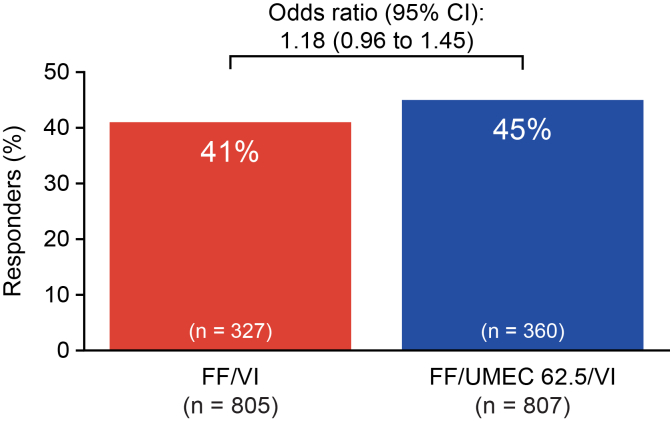
Proportion of responders at weeks 21 to 24.∗ A total of 13% (n = 102) of patients in the FF/VI group and 12% (n = 95) in the FF/UMEC 62.5/VI group were classified as nonresponders because of missing data. If E-RS total score was missing at baseline, patients were excluded from the analysis. ∗Patients with analyzable data at weeks 21 to 24.

There was a greater proportion of responders with FF/UMEC 62.5/VI versus FF/VI (45% [n = 360] vs 41% [n = 327]) at weeks 21 to 24, and patients in the FF/UMEC 62.5/VI group had numerically greater odds of being a responder (≥2-point reduction in score) compared with the FF/VI group (odds ratio, 1.18 [95% CI, 0.96 to 1.45]) (Fig 3).

### Impact of baseline T2 biomarker status on treatment response (*post hoc* analysis)

**Fig 4:**
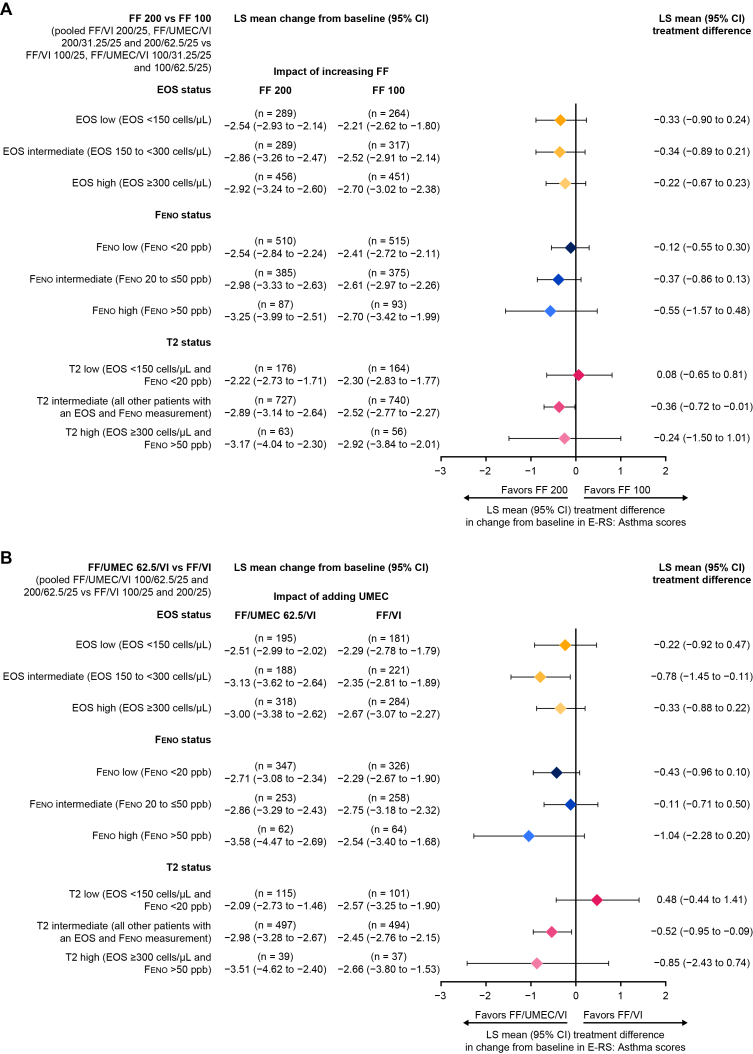
**A** and **B,** Impact of increasing the FF dose (Fig 4, *A*) and adding UMEC (Fig 4, *B*) on E-RS: Asthma total scores by T2 biomarker status at weeks 21 to 24∗ (*post hoc* analysis). ∗Patients with analyzable data at weeks 21 to 24.

The association between baseline T2 biomarker status and the effect of doubling the FF dose (pooled FF 200 μg–containing treatments vs FF 100 μg–containing treatments) or adding UMEC 62.5 μg to FF/VI (pooled UMEC 62.5 μg–containing treatments vs pooled FF/VI) on E-RS: Asthma total score at weeks 21 to 24 is shown in Fig 4. Doubling the FF dose generally led to numerical improvements in E-RS: Asthma total score across the range of biomarker thresholds. These improvements were similar regardless of EOS status at baseline. However, a greater treatment effect from doubling the FF dose was seen in patients with Feno-intermediate (LS mean [95% CI] treatment difference: −0.37 [−0.86 to 0.13]) and Feno-high (−0.55 [−1.57 to 0.48]) than Feno-low (−0.12 [−0.55 to 0.30]) status at baseline and in patients with T2-intermediate (−0.36 [−0.72 to −0.01]) and T2-high (−0.24 [−1.50 to 1.01]) than T2-low (0.08 [−0.65 to 0.81]) status at baseline, although the number of patients in Feno-high and T2-high subgroups were low, limiting interpretability (Fig 4, *A*).

Adding UMEC 62.5 μg to FF/VI led to improvements in E-RS: Asthma total scores across the range of biomarker thresholds, with the exception of patients with T2-low status, for whom FF/VI had a greater treatment effect (Fig 4, *B*). The treatment effect was greater in patients with Feno-high (LS mean [95% CI] treatment difference: −1.04 [−2.28 to 0.20]) versus Feno-low (−0.43 [−0.96 to 0.10]) and T2-high (−0.85 [−2.43 to 0.74]) versus T2-low (0.48 [−0.44 to 1.41]) status at baseline; however, the number of patients with data available in Feno-high and T2-high subgroups was low, again limiting interpretability (Fig 4, *B*). Similar results were seen using unpooled data (see Fig E2 in this article’s Online Repository at www.jaci-global.org).

## Discussion

Among patients with poor asthma symptom control, treatment with either FF/VI or FF/UMEC/VI led to improvements from baseline in E-RS: Asthma total and domain scores. Compared with FF/VI, treatment with FF/UMEC/VI was associated with greater overall numerical improvements in the E-RS: Asthma total score as well as domain scores related to breathlessness and chest symptoms, whereas the odds of achieving the MCID in E-RS: Asthma total score were numerically greater with FF/UMEC/VI versus FF/VI.

The results of this prespecified analysis of E-RS: Asthma data from the CAPTAIN study suggest that the addition of UMEC to FF/VI provides symptom benefits as an alternative to increasing the ICS dose in patients with uncontrolled moderate to severe asthma. Studies have demonstrated that side effects, including increased exacerbations, can occur with medium- to high-dose ICS use,^21^^,^^22^ suggesting that a treatment that provides an alternative to increasing the ICS dose while achieving symptom control is valuable for patient quality of life. The present study also supports previous findings from the CAPTAIN study that showed numerical improvements in ACQ-7 score and greater odds of an ACQ-7 response with FF/UMEC/VI versus FF/VI (nominally statistically significant).^9^ Separately, 2 meta-analyses published in 2021 also reported that modest improvements in asthma control are generally observed with ICS/LAMA/LABA combinations compared with ICS/LABA combinations.^23^^,^^24^ Recently, the concept of clinical remission has been increasingly discussed as an ambitious but attainable treatment goal for all patients with asthma, irrespective of disease severity or treatment history, with 4 key criteria typically proposed, assessed over 12 months or longer: no systemic corticosteroid use, no exacerbations, stabilized or optimized lung function, and symptom control.^1^^,^^25^^,^^26^ Our findings suggest that treatment with FF/UMEC/VI may provide improved symptom control for patients with moderate to severe asthma and attainability of a core component of clinical remission.^1^^,^^17^^,^^18^ Although previous studies have also found improvements in lung function and exacerbation rates with ICS/LAMA/LABA versus ICS/LABA,^9^^,^^23^^,^^24^ further research is required to assess the long-term efficacy of inhaled therapy against a full clinical remission end point, including optimizing treatment based on phenotype to achieve this goal. Potential for personalization of therapy is illustrated by evidence in patients with severe eosinophilic asthma receiving mepolizumab, which found that residual exacerbations had distinct phenotypes, thereby suggesting that more targeted strategies, such as phenotyping using biomarkers, could be preferential to routine oral corticosteroid use or biologic switching.^27^ Following proteomic analysis of sputum samples, a subgroup of patients was identified with elevated expression of a protein cluster containing markers of neutrophil activity, proinflammatory cytokines, and epithelial alarmins who in turn exhibited less favorable treatment outcomes.^28^ This pathophysiological signature may be targetable and treatable by alternative approaches.^28^

In the pooled analysis, improvements in RS-Breathlessness and RS-Chest Symptoms domain scores were greater with FF/UMEC/VI than with FF/VI. Greater improvements were also evident in the RS-Cough and Sputum domain with FF/UMEC/VI versus FF/VI. However, the difference in treatment response was small compared with the other domains, making it difficult to differentiate between the effect of adding UMEC or doubling the FF dose on cough and sputum symptoms. Notably, a 2019 study found that sputum production was significantly more common among patients with chronic cough (with or without asthma) than among patients without chronic cough (with or without asthma).^29^ This suggests that cough and sputum may be linked, with a distinct pathophysiology to other asthma symptoms, which may warrant consideration in treatment decisions. Although data supporting the use of LAMAs to treat cough symptoms are limited, 2 studies in Japanese patients with asthmatic cough resistant to ICS/LABA reported significant improvement in cough-related health-related quality of life and asthma control following treatment with tiotropium, which may have been due to a lowering of the threshold to irritant response, as demonstrated by improvements in capsaicin cough reflex sensitivity scores.^30^^,^^31^ Furthermore, a 2025 randomized controlled trial in Japan reported that in primary care and hospitalized patients with asthma and persistent cough, treatment with FF/UMEC/VI led to significant improvements in cough symptom scores at 6 weeks versus placebo.^32^ Previous research indicates that the cholinergic system regulates the function of goblet cells and mucus production,^33^, ^34^, ^35^ and a systematic review found that anticholinergics such as LAMAs limit the production of mucus in patients with COPD.^36^ Therefore, the reason for the limited response to adding UMEC in the RS-Cough and Sputum domain is unclear. The potential presence of comorbidities such as sinusitis, bronchiectasis, and gastroesophageal reflux disease may have had a masking effect, given they have previously been found to affect mucus production.^37^, ^38^, ^39^, ^40^, ^41^ However, data for these specific comorbidities were not collected in the CAPTAIN study. In addition, ICSs have previously been found to have a role in goblet cell hyperplasia and mucus production,^37^^,^^38^ and shown to improve cough symptoms,^42^, ^43^, ^44^ which may have masked the effect of adding UMEC to FF/VI here.

Asthma management guidelines typically recommend increasing the ICS dose to treat poor asthma control.^1^^,^^6^, ^7^, ^8^ However, patients’ responses to ICSs are variable, and studies have suggested that in some patients, higher ICS doses may be associated with worse asthma control and increased rescue medication use.^45^, ^46^, ^47^ Variability in ICS response may partly be due to patient phenotype, with patients with a T2-low status being less responsive to ICSs.^9^^,^^48^ For example, a UK-based database study found that improvements in exacerbation rates after treatment with medium to high-dose ICSs were more common in patients with high versus low blood EOS counts.^48^ Previous results from the CAPTAIN study have also shown that in patients with uncontrolled moderate to severe asthma not yet considered for biologics, those with a high-T2 status experienced greater improvements in exacerbation rates and lung function than those with a low-T2 status when treated with FF 200 μg– versus 100 μg–containing regimens.^9^ However, in this *post hoc* analysis of the CAPTAIN study, improvements in symptom control from doubling the FF dose were generally similar regardless of T2 status. Therefore, although T2 status is prognostic for future exacerbations and predictive of drug-induced reduction in exacerbation frequency,^9^ the contribution of T2 inflammation to day-to-day symptoms appears less evident. This suggests that additional mechanisms might underlie the symptom burden in patients with asthma, including noneosinophilic (eg, paucigranulocytic) inflammation, impaired lung function, mucus plugging, effects of inhaled steroids on vascular permeability causing fibrinogen transudation, and bronchial hyperresponsiveness associated with mast cell stimulation. Comorbidities such as bronchiectasis, depression, obesity, cytokine release syndrome, and gastroesophageal reflux disease may also contribute to airway damage and symptoms.

Notably, we found that the addition of UMEC to FF/VI had a greater treatment effect than FF/VI alone, across most EOS, Feno, and T2 subgroups. These observations highlight the importance of considering both patient phenotype (eg, inflammation status) and the desired treatment outcome when assessing therapeutic options. This is further illustrated by several studies showing that in patients with uncontrolled severe asthma with an eosinophilic phenotype, the use of biologics reduced exacerbations, improved symptom control, and lowered sputum EOS counts versus comparator treatments,^49^, ^50^, ^51^, ^52^, ^53^ and EOS counts have also been established as a predictor of biologic treatment response.^54^^,^^55^

The use of the E-RS: Asthma tool in this study follows its previous validation in populations with asthma and asthma-COPD overlap.^15^^,^^17^ This tool was shown to be reliable and responsive to changes in asthma symptoms on a daily basis and captures the most important symptoms from a patient perspective.^15^ Indeed, the E-RS: Asthma assesses cough symptoms as part of the combined RS-Cough and Sputum domain, but the ACQ does not include cough and the Asthma Control Test assesses symptoms more generally (wheezing, coughing, shortness of breath, chest tightness, or pain).^18^^,^^56^ Therefore, the E-RS: Asthma may offer both an adjunct and alternative to other asthma symptom assessment tools, which are performed on a less frequent basis, and may provide additional sensitivity. Interestingly, previous studies have reported mixed results regarding the association between different asthma symptom tools and lung function.^12^, ^13^, ^14^ Given the separation of respiratory symptoms into individual domains in the E-RS: Asthma, future research may leverage this to explore any correlation between specific symptoms and spirometry outcomes, which may help define patient phenotypes more responsive to targeted interventions.

The CAPTAIN study has several strengths that should be acknowledged. The study used the E-RS: Asthma to evaluate symptom control, which measures the day-to-day impact of symptoms with outcomes that are relevant and important to patients.^15^ An additional strength of this study was its design: eligible patients first entered a 3-week run-in period, during which they received FP/SAL 250/50 μg and were then assessed for eligibility on the basis of the specific enrollment criteria. The inclusion of a run-in period eliminated the impact of treatment adherence on the study’s findings, meaning that the results show the actual effects of the study treatment. Furthermore, the multicenter, multicountry design ensured a broad representation of patient demographic characteristics, and the lack of an exacerbation eligibility requirement improved study generalizability.

However, the results of this analysis should also be interpreted in the context of its limitations. Although a benefit of the CAPTAIN run-in period was that it allowed the inclusion of patients with uncontrolled symptomatic asthma despite requiring daily ICS/LABA maintenance therapy, representing a study population at GINA steps 4 and 5, this approach is not reflective of real-world practice. As such, the real-world treatment effects of adding UMEC to FF/VI may be greater than those observed in the CAPTAIN study. An additional consideration is the break in the statistical hierarchy of the CAPTAIN study, which prevented testing for statistical significance, meaning that the analyses reported here were descriptive in nature. *Post hoc* analyses according to T2 biomarker status were also limited by low number of patients in Feno-high and T2-high subgroups. In addition, limitations of the E-RS: Asthma include the combination of cough and sputum symptoms into a single domain, which may limit analysis of the effect of treatment on individual symptoms. Furthermore, there is less real-world experience of the use of the E-RS: Asthma compared with the E-RS: COPD and with earlier tools such as the ACQ and the Asthma Control Test. Although the E-RS has been validated in patients with asthma, extension of this validation to a more ethnically and culturally diverse population would further reinforce its generalizability.^15^

### Conclusion

Treatment with either FF/VI or FF/UMEC/VI resulted in improvements in E-RS: Asthma total and domain scores, whereas the addition of UMEC to FF/VI led to numerical improvements in E-RS: Asthma scores and numerically greater odds of having a response compared with FF/VI. Treatment response was also generally greater with FF/UMEC/VI than with FF/VI, regardless of T2 inflammation status. These results suggest that patients enrolled in the CAPTAIN study, representing those with symptomatic asthma but not preselected on the basis of a high exacerbation rate, may benefit from interventions with optimized treatment, such as adding a LAMA to ICS/LABA, although additional research is needed to further explore these findings.

## Disclosure statement

This study was funded by GSK (GSK study no. 205715; NCT02924688).

Disclosure of potential conflict of interest: E. Pizzichini was an employee of GSK at the time of the study and owned stocks/shares in GSK. G. Brusselle has received speaker fees from and served on advisory boards for AstraZeneca, Boehringer Ingelheim, Chiesi, GSK, Novartis, and Sanofi. J. Crawford, D. Slade, L. Yuanita, and A. Moore are employed by GSK and hold financial equities in GSK. H. Inoue has received institutional grants or personal fees for consulting or lecturing from AstraZeneca, Boehringer Ingelheim, GSK, Kyorin, Novartis, Omron, Sanofi, and Teijin-Pharma. H. A. M. Kerstjens has received research or educational grants and served on advisory boards for Boehringer Ingelheim, GSK, and Novartis and has served on advisory boards for Chiesi and AstraZeneca, all paid to their institution. J. Oppenheimer has served on adjudication committees or data and safety monitoring boards for AstraZeneca, GSK, Novartis, and Sanofi-Regeneron; and has received grants and personal fees from GSK. A. Papi has received research grants from Chiesi, AstraZeneca, GSK, Boehringer Ingelheim, Teva, and Sanofi; has received consulting fees from Chiesi, AstraZeneca, GSK, Novartis, Sanofi, IQVIA, Avillion, Elpen Pharmaceuticals, and MSD; has received payment or honoraria for lectures, presentations, speakers bureaus, manuscript writing, or educational events from Chiesi, AstraZeneca, GSK, Boehringer Ingelheim, Menarini, Novartis, Zambon, Mundi Pharma, Teva, Sanofi, Edmond Pharma, and IQIVA; and has participated on a data safety monitoring board or advisory board for Chiesi, AstraZeneca, GSK, Novartis, Sanofi, IQVIA, Avillion, Elpen Pharmaceuticals, and MSD. I. D. Pavord has received speaker’s fees, payments for organizing education events, honoraria for attending advisory panels, sponsorship to attend international scientific meetings, research grants or payments to support Food and Drug Administration approval meetings from Aerocrine, Almirall, AstraZeneca, Boehringer Ingelheim, Chiesi, Circassia, Roche-Genentech, GSK, Knopp, Merck, Novartis, Sanofi-Regeneron, and Teva; has acted as an expert witness for a patent dispute involving AstraZeneca and Teva; is a co-patent holder for the Leicester Cough Questionnaire; and has received payments for use of the Leicester Cough Questionnaire in clinical trials from Bayer, Insmed, and Merck.

## Supplementary data

Supplementary Figs E1 and E2
