# Supplementary material for: Effect of once-daily ICS/LAMA/LABA triple therapy versus ICS/LABA on respiratory symptoms (E-RS: Asthma): Analysis of the phase IIIA CAPTAIN trial
Source: J Allergy Clin Immunol Glob. 2026 Jun 1;5(5):100741. doi: 10.1016/j.jacig.2026.100741 (PMC13356626; doi:10.1016/j.jacig.2026.100741)
Supplement: Supplementary Figs E1 and E2 [file mmc1.docx]

**Effect of once-daily ICS/LAMA/LABA triple therapy versus ICS/LABA on respiratory symptoms (E-RS: Asthma) in the CAPTAIN trial**

Emilio Pizzichini, MD, Guy Brusselle, MD, Jodie Crawford, MSc, Hiromasa Inoue, MD,
Huib A.M. Kerstjens, MD, John Oppenheimer, MD, Alberto Papi, MD, Ian D. Pavord, FMedSci,
David Slade, MD, Liza Yuanita, MD, Alison Moore, PhD

**Supplementary Material**

***Supplementary Figure 1. Unpooled E-RS: Asthma total and domain scores at
 Weeks 21–24* (post hoc analysis^†^)***


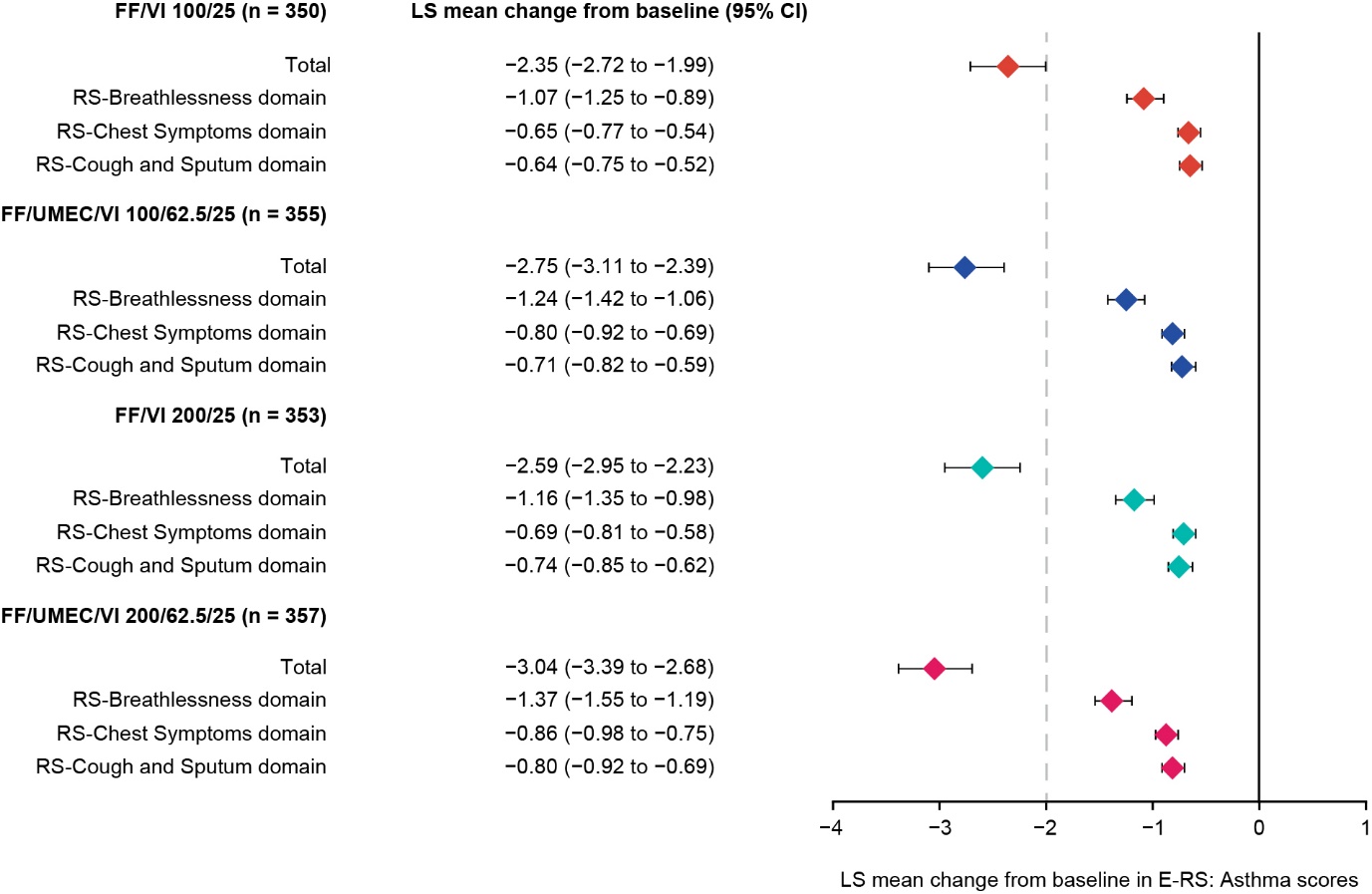


*Patients with analyzable data at Weeks 21–24; ^†^unpooled analyses of E-RS: Asthma total scores were prespecified; analyses of individual domains were post hoc.

The MCID for improvement in E-RS: Asthma total score of ≥2.0 units (decrease) from baseline at Weeks 21–24 is indicated by the vertical dashed line.

CI, confidence interval; E-RS, Evaluating Respiratory Symptoms; FF, fluticasone furoate; LS, least squares; MCID, minimum clinically important difference; RS, Respiratory Symptoms; UMEC, umeclidinium; VI, vilanterol.

***Supplementary Figure 2. Unpooled E-RS: Asthma total scores by T2 biomarker status at Weeks 21–24*: (A) EOS, (B) FeNO and (C) T2 combined (post hoc analysis)***


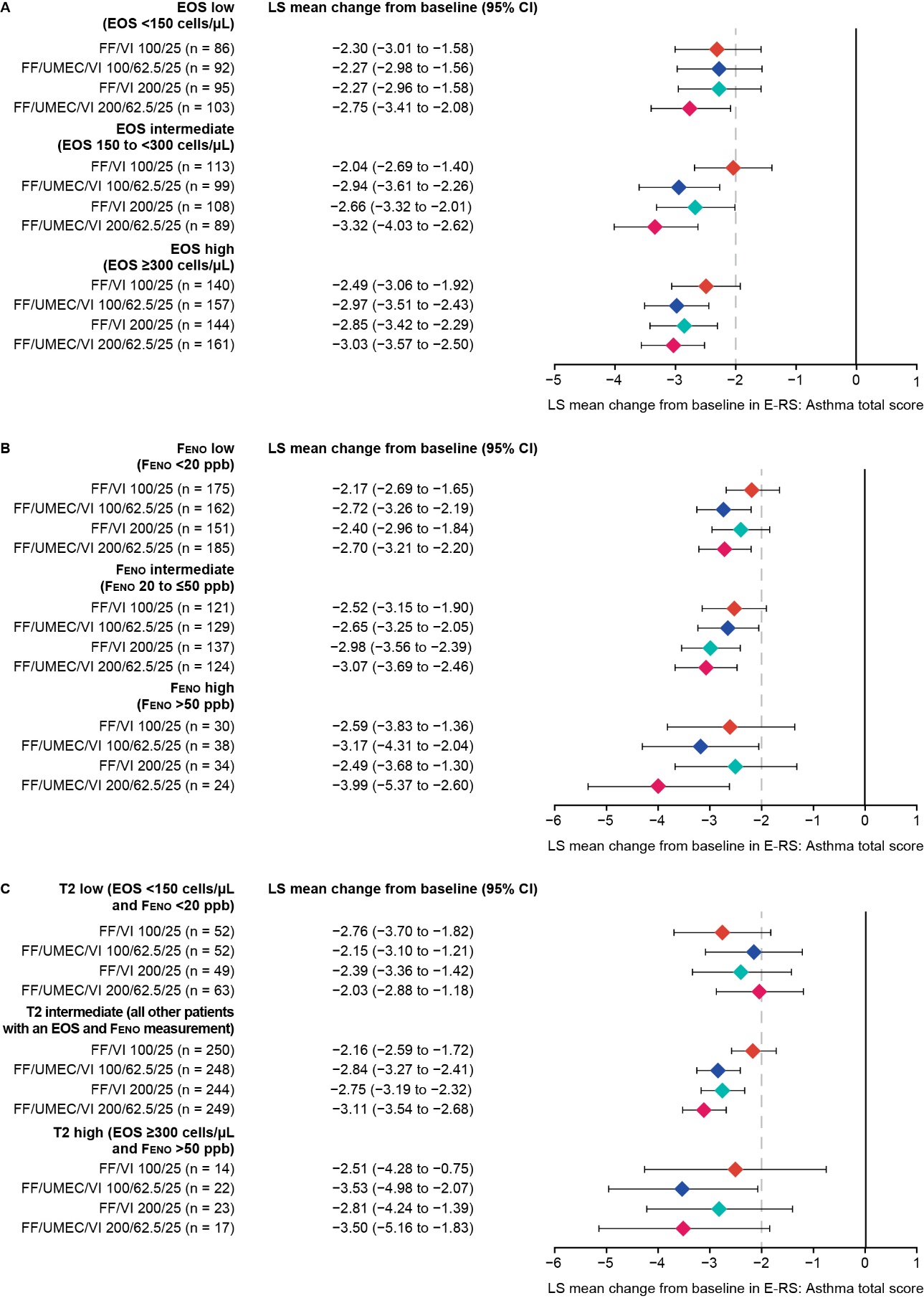


*Patients with analyzable data at Weeks 21–24.

The MCID for improvement in E-RS: Asthma total score of ≥2.0 units (decrease) from baseline at Weeks 21–24 is indicated by the vertical dashed line.

CI, confidence interval; EOS, eosinophil; E-RS, Evaluating Respiratory Symptoms; FeNO, fractional exhaled nitric oxide; FF, fluticasone furoate; LS, least squares; MCID, minimum clinically important difference; ppb, parts per billion; RS, Respiratory Symptoms; T2, type 2; UMEC, umeclidinium; VI, vilanterol.
